# Trait emotional intelligence profiles of professionals in Kuwait

**DOI:** 10.3389/fpsyg.2023.1051558

**Published:** 2023-02-17

**Authors:** Nasser N. Hasan, Konstantinos V. Petrides, Laura Hull, Fawziyah Hadi

**Affiliations:** ^1^Department of Clinical, Educational, and Health Psychology, University College London, London, United Kingdom; ^2^Department of Educational Psychology, College of Education, Kuwait University, Kuwait City, Kuwait; ^3^London Psychometric Laboratory, University College London, London, United Kingdom; ^4^Department of Population Health Sciences, Centre for Academic Mental Health, Bristol Medical School, University of Bristol, Bristol, United Kingdom

**Keywords:** trait emotional intelligence, TEIQue-SF, organizational commitment, job satisfaction, job attitudes, job performance

## Abstract

Trait emotional intelligence concerns people’s perceptions of their emotional world. Our aims for this study are to examine (a) the trait emotional intelligence (EI) profiles across different professions in Kuwait; (b) the incremental validity of trait EI in predicting job performance; and (c) the relationship between trait EI, job attitudes, and job performance. The sample comprised 314 professionals in Kuwait in seven different professions: Bankers, Engineers, Healthcare providers, Lawyers, Military, Policemen, and Teachers. Firstly, the results showed that the Military scored the lowest global trait emotional intelligence and three of four factors. Secondly, the results showed that global trait EI incrementally predicted job performance over job attitudes in Policemen and Engineers but not in other professions. Lastly, the results showed that job attitudes partially mediated the relationship between trait EI and job performance. These findings call for the importance of trait emotional intelligence trainings for professionals in Kuwait as it affects important job-related variables. The limitations of this study and the directions for future studies have been discussed.

## Introduction

Employees’ emotions play a crucial part in the workplace (Ashkanasy and Dorris, 2017). Evidence from the field of Organizational Psychology and Behavior suggests that positive emotions influence workplace success-related variables, such as creativity, work engagement, coping, teamwork, and collaboration (Diener et al., 2020). Further evidence in the form of meta-analysis by Shockley et al. (2012) revealed that negative emotions were associated with harmful organizational behaviors. In fact, earlier findings from the decision-making field showed that people’s behavior is determined by the emotions they expect to experience in the future or those they have experienced previously (Mellers et al., 1999). These effects have also been investigated with reference to trait emotional intelligence theory to which we now turn (Sevdalis et al., 2007).

### Trait emotional intelligence

In simple words, trait emotional intelligence (EI) is concerned with people’s perceptions of their emotional abilities and is assessed through self-report questionnaires. The key difference between trait EI and ability EI is how they operationalize. The ability EI is based on the maximum performance tests, such as IQ tests, with correct and wrong answers. It is troublesome because of the subjectivity of emotional experiences. Contrarily, the trait EI operationalization is straightforward because it includes self-perceptions and dispositions aligned with the subjective nature of emotions. Consequently, the number of trait EI models and measures exploded, providing an impression that it is an easy business. However, anyone with basic psychometrics knowledge knows that it is not.

Petrides and Furnham (2000, 2001) content-analyzed notable EI model and relevant constructs to derive their first sampling domain that specifically underlies the trait EI construct. This step was crucial to operationalize any psychological construct (Cattell, 1973). Their sampling domain comprised 15 facets: adaptability, assertiveness, emotional appraisal toward self and others, emotion expression, emotion management toward others, emotion regulation, low impulsiveness, relationship skills, self-esteem, self-motivation, social competence, stress management, trait empathy, trait happiness, and trait optimism. This model is the most scientifically acceptable trait EI model compared to other models because the above step was bypassed when the construct was defined in the earlier models (e.g., Salovey and Mayer, 1990; Bar-On, 2004).

Accordingly, Petrides et al. (2007) defined trait EI as a combination of emotional perceptions that may be measured using questionnaires and rating scales such as the Trait Emotional Intelligence Questionnaire (TEIQue; Petrides, 2009). The TEIQue offers a comprehensive operationalization for the trait EI model providing full coverage of its sampling domain. Accordingly, it is the most appropriate tool to investigate the emotional profiles of different professions and occupations, as has been argued in previous relevant studies (Petrides et al., 2022).

Several studies found that trait EI predicts many job-related variables such as job performance (O'Boyle et al., 2011; Joseph et al., 2015; Li et al., 2018), job satisfaction (Platsidou, 2010; Anari, 2012; Ignat and Clipa, 2012; Schutte and Loi, 2014; Hubscher-Davidson, 2016; Li et al., 2018), and organizational commitment (Nikolaou and Tsaousis, 2002; Petrides and Furnham, 2006; Salami, 2008). In fact, Sackett et al. (2021) showed that trait EI was the best personality-related predictor of job performance in their meta-analytic study. Yet these either focused exclusively on a single professional group (e.g., teachers) or pooled together multiple professions without regard to their unique characteristics. This study contributes to the existing body of the trait EI literature by including different and previously uninvestigated professions.

### Job attitudes and job performance

Job performance is one of the most researched concepts within organizational settings, and one of the key variables included in our study. Motowidlo (2003) viewed job performance as a set of behaviors carried out by an individual that the organization expects in a period of time. In one meta-analytic study, O'Boyle et al. (2011) found that trait EI is a very strong predictor of job performance with clear incremental validity over cognitive ability and the Five Factors of personality. In fact, Sackett et al. (2021) found, in a recent meta-analytic study, that trait EI is the best personality-related predictor of job performance.

Other job-related variables included in our study are job satisfaction, organizational commitment, and job attitudes. Job satisfaction can be viewed as how people feel about their job and several aspects related to it (Spector, 1997). While organizational commitment is viewed as people’s psychological bond to the organization and how they persist in sacrificing for this organization (Allen and Meyer, 1990).

Judge and Kammeyer-Mueller (2012) argued that job attitude is a multifaceted construct composed of several job-related attitudes such as job satisfaction and organizational commitment. Thus, job attitudes can be viewed as a hierarchical evaluation of one’s feelings toward their job (i.e., job satisfaction) and one’s attachment to their job (i.e., organizational commitment). For this, we will view job attitudes as a latent variable represented by both job satisfaction and organizational commitment throughout this project.

### The relationship between trait emotional intelligence and job attitudes

Miao et al. (2017) studied the relationship between EI and job attitudes concepts (e.g., job satisfaction and organizational commitment). Their meta-analytic study found that self-reported trait EI was positively related to job attitudes. Thus, participants with higher trait EI generally tend to have higher job satisfaction and organizational commitment.

Researchers started their investigation on the causal relationship between job attitudes and job performance in the 70s (e.g., Siegel and Bowen, 1971; Wanous, 1974; Sheridan and Slocum Jr, 1975). Since then, most studies have shown that job performance and positive job attitudes (e.g., job satisfaction and organizational commitment) were positively correlated. Yet, the causal relationship between them was inconclusive. Explicitly, does job performance increase job attitudes, or it is the other way around? Riketta (2008) conducted a meta-analytic regression to answer this question. The results of his study favored the idea that positive job attitudes influenced job performance, but not the other way around. Therefore, we will retest this view in the present study.

### The case in Kuwait

None of the foregoing meta-analyses have included results from Kuwait. Two reasons may potentially explain that. One is related to the fact that relevant studies may have been published in Arabic only, making them inaccessible to English researchers. The other reason could be the lack of studies in Kuwait. Either way, there is a noticeable lack of studies in Kuwait published in English that can be globally reached by interested people.

As in most countries, two job providers exist in Kuwait: the government and the private sectors. The Central Statistical Bureau of Kuwait (2021) reported that three-quarters of the employees in the government sector are Kuwaitis, while they only represent 4.5% of the employees in the private sector (The Central Statistical Bureau of Kuwait, 2014a, b). This discrepancy can be due to the salary paid by each sector compared to the job nature, working hours, and job security. Excluding the military sector from their report, CBS showed that 56% of the employees in the government sector are females, in which most of them are working in the Ministry of Education. Further, the report showed that most employees in the government sector are between 25 and 44 years old. The results were similar in the private sector.

In this study, we are contributing to the literature by studying the relationship between trait emotional intelligence, job attitudes, and job performance in different professions within Kuwait. Even more, we will discuss how the findings in Kuwait are different than those in other countries. We will also present a starting-up trait EI profile for each profession for future researchers who wish to investigate these profiles for any purpose.

## Materials and methods

### Design and procedure

We applied a survey design to achieve our study’s objectives. We used non-proportional quota sampling method and approached several Kuwaiti organizations (governmental and private sectors) to collect data from their employees. Participants were invited to provide their voluntary consent and then complete the measures. Participants did not provide any personal information that allow researchers or organizations to identify them. Data collection was both online and *via* paper and pencil. All participations were voluntary, and no compensation was offered to both participants and organizations.

### Participants

Three hundred and fourteen professionals participated in the study with a mean age of 33.62 years (SD = 12.24 years). The sample comprised 174 males and 135 females, while 5 participants preferred not to reveal their gender. Two hundred ninety-two participants were Kuwaitis; the rest were non-Kuwaitis, mainly from other Arab-region countries. The sample also comprises 154 married participants, 126 single participants, and 26 divorced participants, while the rest preferred not to reveal their current marital status.

The following professions were represented in our sample: Bankers (*n* = 36), Engineers (*n* = 72), Healthcare providers (*n* = 33), Lawyers (*n* = 35), Military (*n* = 33), Policemen (*n* = 39), and Teachers (*n* = 66). Gender and age information for each profession is reported in Table 1.

**Table 1:** Gender and age information for each profession represented in our sample.

Profession	Gender			Age (Years)
	Males	Females	PNS	Mean (SD)
Bankers (*n* = 36)	21	15	0	31.89 (17.39)
Engineers (*n* = 72)	31	38	3	33.26 (10.99)
Healthcare providers (*n* = 33)	15	17	1	36.85 (7.99)
Lawyers (*n* = 35)	16	19	0	32.89 (9.55)
Military (*n* = 33)	33	0	0	35.12 (11.84)
Policemen (*n* = 39)	35	3	1	34.36 (15.74)
Teachers (*n* = 66)	23	43	0	32.55 (11.12)
Overall sample (*n* = 314)	174	135	5	33.62 (12.24)

PNS = prefer not to say, SD = standard deviation.

### Measures

#### Job performance

This was measured with a single self-report question as in Petrides et al. (2022). Participants were asked explicitly to evaluate how good they were in their job using a single item: “How do you evaluate yourself in doing your job out of 100?”

#### Trait emotional intelligence questionnaire-short form (TEIQue-SF)

The TEIQue-SF is a 30-item inventory (e.g., “On the whole, I’m pleased with my life;” “Others admire me for being relaxed”) providing comprehensive coverage of the sampling domain of trait EI in adults (Petrides, 2009). The items are responded to a 7-point Likert scale ranging from “completely disagree” to “completely agree.” All TEIQue instruments are available, free of charge, for research purposes from www.psychometriclab.com. In this study, we used the Kuwaiti-Arabic adaptation of TEIQue-SF (Hasan et al., 2023), which has shown robust psychometric properties in Kuwaiti samples. Cronbach’s alpha value for the TEIQue-SF in our study was.88.

#### Minnesota satisfaction questionnaire (MSQ)-short form

The MSQ is a 20-item inventory (e.g., On my present job, this is how I feel about: “being able to keep busy all the time;” “being able to do things that do not go against my conscience”) developed by Weiss et al. (1967). It measures job satisfaction based on a 5-point Likert response scale, ranging from “very dissatisfied” to “very satisfied.” Al-Mutairi et al. (2017) used the MSQ with a Kuwaiti sample and reported a coefficient alpha of.93 in their study. Cronbach’s alpha value for the MSQ in our study was.90.

#### Organizational commitment questionnaire (OCQ)

The OCQ is a 15-item inventory (e.g., “I am proud to tell others that I am part of this organization;” “I find that my values and the organization’s values are very similar”) measuring employee’s organizational commitment. It was developed by Mowday et al. (1979). The items are responded to a 7-point Likert scale, ranging from “strongly disagree” to “strongly agree.” Al-Ajmi (2006) used the OCQ with a Kuwaiti sample and reported a coefficient alpha of 0.85 in his study. Cronbach’s alpha value for the OCQ in our study was 0.87.

### Data analysis plan

**Figure 1:**
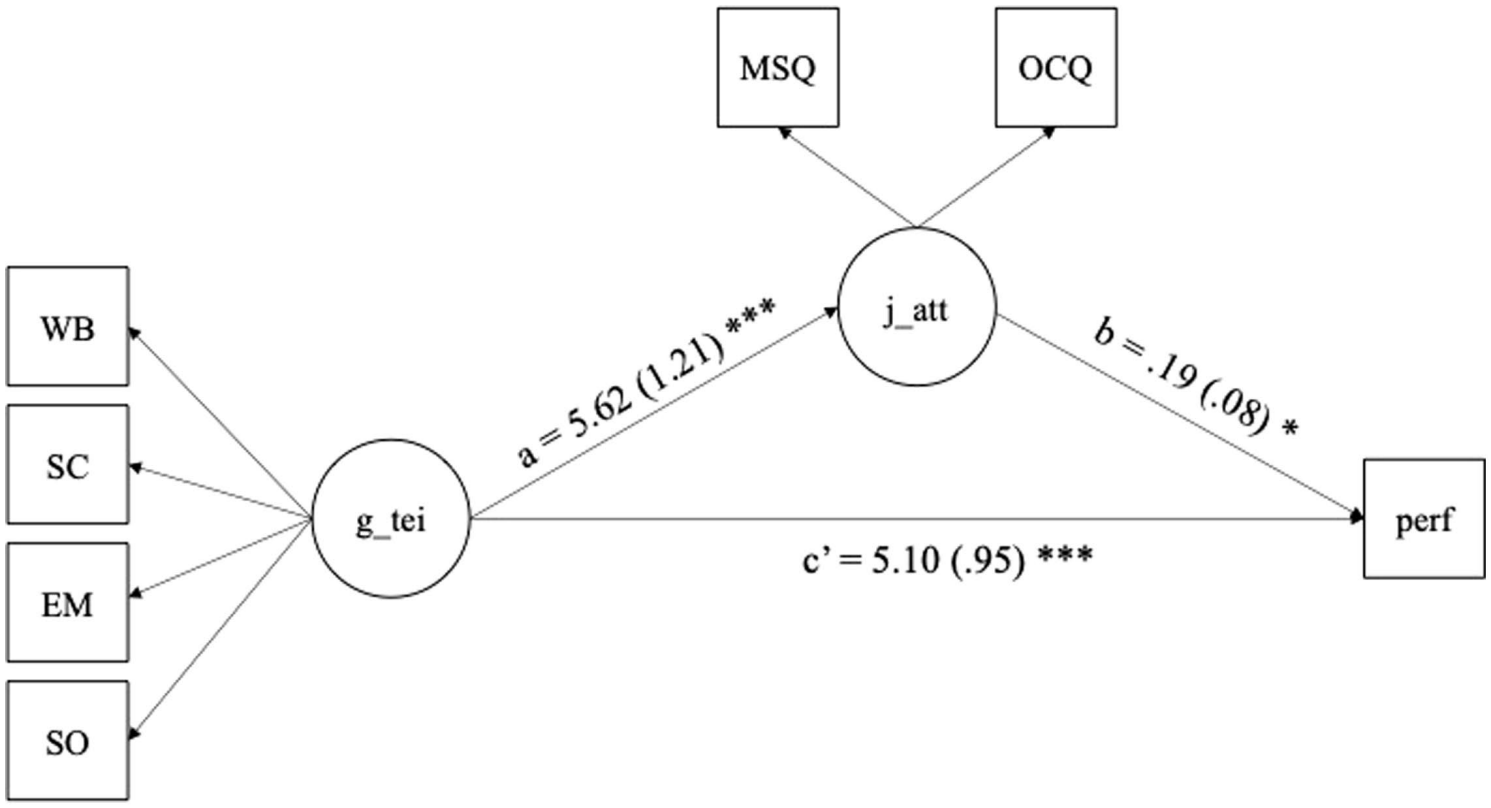
The Mediation Model. ^a^Effect of trait EI on job attitudes. ^b^Effect of job attitudes on job performance. ^c’^Direct effect of trait EI on job performance. Indirect effect of trait EI on job performance (rounded to two decimals) = a x b = 5.62 x .19 = 1.07. The number before the brackets is the estimated effect (associated standard error) rounded to 2 decimal places, g_tei = Global trait EI, j_att = Job attitudes, perf = Job Performance, MSQ = Job satisfaction score, OCQ = Organizational commitment score, WB = Well-being, SC = Self-control, EM = Emotionality, SO = Sociability. *p < 0.05; **p < 0.01; ***p < 0.001.

Descriptive statistics and Cronbach’s alpha estimates were obtained using SPSS (IBM Corp. Released, 2020). Further, we discussed earlier how trait EI and job attitudes affect job performance. We also discussed how trait EI and job attitudes were related. Accordingly, we proposed the model shown in Figure 1, which we will test through Structural Equation Modeling (SEM) using Mplus (Muthén and Muthén, 1998-2017). Model parameters will be estimated with the robust maximum likelihood estimator (MLR) to deal with deviations from normality. This will offer evidence for the criterion validity of the TEIQue-SF.

Hair et al. (2010) advised researchers to consider both sample size and model complexity when choosing the appropriate fit cutoff. That is, research with larger samples and less complex models requires stricter criteria. Clearly, they explicitly argued that neither 0.90 nor 0.95 (as an acceptable cutoff for CFI) are “magic” values to distinguish between good and bad models. Even more, they provided characteristics of different fit indices representing acceptable fits for different model situations. For instance, they would expect a CFI of.92 or above, SRMR < 0.09, and RMSEA < 0.08, for a situation similar to the present study.

We will also examine the incremental validity of trait EI over job attitude variables in predicting job performance across different professions. Specifically, job performance will be regressed on job satisfaction and organizational commitment (Step 1), with the global trait EI score added subsequently to the model (Step 2).

One-way ANOVA and MANOVA will be used to compare the trait EI profile (i.e., global trait EI and the four factors) for each profession. Accordingly, a *post-hoc* analysis will be carried out using Tukey HSD *post-hoc* tests (if the assumption of homogeneity of variance is met) or Games-Howell *post-hoc* tests (if the assumption is violated).

## Results

The descriptive statistics for all key variables in our study are presented in Table 2.

**Table 2:** Descriptive statistics for the key variables in the study across professions.

Profession	Global trait EI				MSQ				OCQ			
	Range^a^	M (SD)	Skew	Kurt	Range^b^	M (SD)	Skew	Kurt	Range^c^	M (SD)	Skew	Kurt
Bankers (*n* = 36)	3.80–6.67	5.11 (0.66)	0.13	−0.23	48.00–99.00	76.83 (13.26)	−0.56	−0.28	42.00–99.00	66.53 (11.88)	0.02	0.83
Engineers (*n* = 72)	3.67–7.00	5.10 (0.83)	0.04	−0.79	23.00–97.00	67.25 (14.79)	−0.72	0.95	43.00–101.00	63.78 (10.90)	0.81	1.01
Healthcare providers (*n* = 33)	3.90–6.47	5.14 (0.62)	0.04	−0.12	26.00–92.00	69.45 (15.58)	−1.02	0.64	15.00–87.00	62.91 (12.33)	−1.69	6.3
Lawyers (*n* = 35)	3.97–6.77	5.48 (0.66)	−0.09	−0.55	49.00–100.00	74.86 (12.74)	0.03	−0.42	44.00–84.00	65.31 (9.37)	−0.11	−0.24
Military (*n* = 33)	3.63–6.53	4.55 (0.80)	1.07	−0.06	59.00–88.00	71.24 (8.75)	0.48	−0.93	53.00–88.00	65.03 (7.81)	0.76	0.83
Policemen (*n* = 39)	2.40–6.43	5.19 (0.88)	−0.79	1.13	51.00–97.00	76.77 (10.97)	−0.55	0.19	50.00–102.00	66.69 (10.06)	1.14	2.87
Teachers (*n* = 66)	3.50–6.77	5.20 (0.81)	−0.03	−0.94	38.00–100.00	69.23 (14.87)	0.17	−0.72	45.00–93.00	61.86 (10.25)	0.81	0.97

a The theoretical range is 1.00–7.00.

b The theoretical range is 20.00–100.00.

c The theoretical range is 15.00–105.00.

EI = Emotional Intelligence, MSQ = Minnesota Satisfaction Questionnaire, OCQ = Organizational Commitment Questionnaire, M = Mean, SD = Standard deviation, Skew = Skewness, Kurt = Kurtosis.

### The relationship between trait emotional intelligence and job-related variables

Overall sample and gender-based correlations are presented in Table 3. Pearson’s correlations between the three variables were all statistically significant at *p* < 0.01 as follows: TEIQue-SF and job performance (*r* = 0.38), *TEIQue*-SF and MSQ (*r* = *0*.29), TEIQue-SF and OCQ (*r* = *0*.15), and MSQ and OCQ (*r* = 0.49). The significant correlations between TEIQue-SF and the three job-related measures (Job performance, MSQ, and OSQ) support the criterion validity of the TEIQue-SF in the Kuwaiti professionals’ sample.

**Table 3:** Gender-based Cronbach’s *α* for key variables in the study.

		Overall sample (*N* = 314)	Males (*N* = 174)	Females (*N* = 135)
TEIQue-SF				
	Global trait EI	0.88	0.90	0.85
	Wellbeing	0.74	0.74	0.72
	Self-control	0.54	0.55	0.53
	Emotionality	0.58	0.59	0.58
	Sociability	0.69	0.71	0.65
MSQ		0.90	0.89	0.91
OCQ		0.87	0.85	0.89

Mediation model 1 proposed that job attitudes will mediate the relationship between trait EI and job performance. Model fit values were acceptable for the proposed model (Figure 1), CFI = 0.94, RMSEA = 0.09 [90% CI: 0.06–0.12], SRMR = 0.05, based on Hair et al. (2010) demonstration for a good model.

The SEM results indicated that job attitudes significantly predict job performance, *β* = 0.19, *SE* = 0.08, *p* < 0.05, accounting for approximately 11.2% of the variance, *R^2^* = 0.112; trait EI significantly predicted both job attitudes, *β* = 5.62, *SE* = 1.21, *p* < 0.001, as well as job performance, *β* = 5.10, *SE* = 0.95, *p* < 0.001. The latter constitutes the direct effect of trait EI on job performance in our model. The indirect effect, tested using bootstrapped standard errors, was also significant, *β* = 1.07, *SE* = 0.45, *p* < 0.05. These findings suggest that job attitudes partially mediated the relationship between trait EI and job performance. The total effect of trait EI on job performance was 6.17.

### Incremental validity of trait emotional intelligence

Hierarchical regression analysis summaries are shown in Table 4. In this analysis, job satisfaction and organizational commitment were entered together at step 1, while trait EI was entered on its own at step 2. We now briefly present the results separately for each profession.

**Table 4:** Hierarchical multiple linear regression results.

	Bankers		Engineers		Healthcare Providers		Lawyers		Military		Policemen		Teachers	
Step 1	*F*(2, 33) = 3.29, *R*^2^ = 0.166, adj. *R*^2^ = 0.116		*F*(2, 67) = 3.93^*^, *R*^2^ = 0.105, adj. *R*^2^ = 0.078		*F*(2, 30) = 0.23, *R*^2^ = 0.015, adj. *R*^2^ = −0.05		*F*(2, 32) = 1.11, *R*^2^ = 0.065, adj. *R*^2^ = 0.007		*F*(2, 30) = 5.53^*^, *R*^2^ = 0.269, adj. *R*^2^ = 0.221		*F*(2, 35) = 2.64, *R*^2^ = 0.131, adj. *R*^2^ = 0.082		*F*(2, 63) = 11.92^*^, *R*^2^ = 0.275, adj. *R*^2^ = 0.252	
Step 2	*F*(3, 32) = 2.76, *R*^2^ = 0.205, adj. *R*^2^ = 0.131		*F*(3, 66) = 5.67^*^, *R*^2^ = 0.205, adj. *R*^2^ = 0.169		*F*(3, 29) = 0.92, *R*^2^ = 0.087, adj. *R*^2^ = −0.008		*F*(3, 31) = 1.10, *R*^2^ = 0.096, adj. *R*^2^ = 0.009		*F*(3, 29) = 4.01^*^, *R*^2^ = 0.293, adj. *R*^2^ = 0.220		*F*(3, 35) = 4.23, *R*^2^ = 0.272^*^, adj. *R*^2^ = 0.207		*F*(3, 62) = 8.95, *R*^2^ = 0.302, adj. *R*^2^ = 0.268	
Δ*R*^2^	0.039		0.100^*^		0.071		0.031		0.024		0.140^*^		0.028	
Predictor	β	t	β	t	β	t	β	t	β	t	β	t	β	t
(Step 1)
MSQ	0.09	0.4	0.28	2.09^*^	0.14	0.65	0.28	1.44	0.56	3.32^*^	0.35	2.13^*^	0.21	1.73
OCQ	0.35	1.62	0.08	0.62	−0.11	−0.51	−0.06	−0.31	−0.18	−1.06	0.02	0.14	0.38	3.1^*^
(Step 2)
MSQ	−0.4	−0.16	0.25	1.97	0.21	1.01	0.2	0.97	0.37	1.47	0.23	1.4	0.12	0.91
OCQ	0.41	1.87	0.05	0.36	−0.18	−0.85	−0.09	−0.46	−0.14	−0.79	0.08	0.53	0.38	3.09^*^
global trait EI	0.22	1.26	0.32^*^	2.88^*^	0.28	1.5	0.2	1.04	0.23	0.99	0.39	2.56^*^	0.19	1.57

β = Standardized Beta estimate, MSQ = Job satisfaction score, OCQ = Organizational commitment score, EI = Emotional Intelligence.

* *p* < 0.05.

At step 1 for Bankers, the model predicted 16.6% of the variance in job performance (*F*_(2, 33)_ = 3.29, *p* = 0.05). Both job attitude variables entered in step 1 were not significant predictors of job performance. Trait EI predicted 3.90% of unique variance in job performance after controlling for job attitude variables in step 2 (*F*_Change (1, 32)_ = 1.59, *p* = 0.22). However, trait EI was not a significant predictor of job performance.

For Engineers, the model predicted 10.5% of the variance in job performance at step 1 (*F*_(2, 67)_ = 3.93, *p* > 0.05), MSQ was found to be a significant positive predictor of job performance (β_MSQ_ = 0.28, *t* = 2.09, *p* < 0.05). At step 2, trait EI was found to be the only significant positive predictor of job performance (β_trait EI_ = 0.32, *t* = 2.88, *p* < 0.05). Trait EI predicted a significant 10.0% of unique variance in job performance after controlling for job attitude variables (*F*_Change (1, 66)_ = 8.28, *p* < 0.05).

For Healthcare Providers, the model predicted 1.5% of the variance in job performance at step 1 (*F*_(2, 30)_ = 0.23, *p* = 0.79). Both job attitude variables entered in step 1 were not significant predictors of job performance. Trait EI predicted 7.1% of unique variance in job performance after controlling for job attitude variables in step 2 (*F*_Change (1, 29)_ = 2.26, *p* = 0.14). However, trait EI was not a significant predictor of job performance.

For Lawyers, the model predicted 6.5% of the variance in job performance at step 1 (*F*_(2, 32)_ = 1.11, *p* = 0.34). Both job attitude variables entered in step 1 were not significant predictors of job performance. Trait EI predicted 3.1% of unique variance in job performance after controlling for job attitude variables in step 2 (*F*_Change (1, 31)_ = 1.07, *p* = 0.31). However, trait EI was not a significant predictor of job performance.

For Military, the model predicted 26.9% of the variance in job performance at step 1 (*F*_(2, 30)_ = 5.53, *p* < 0.05), MSQ was found to be a significant positive predictor of job performance (β_MSQ_ = 0.56, *t* = 3.32, *p* < 0.05). At step 2, trait EI was not a significant predictor of job performance, after controlling for job attitude variables. Trait EI only predicted 2.40% of unique variance in job performance after controlling for job attitude variables (*F*_Change (1, 29)_ = 0.98, *p* = 0.33).

For Policemen, the model predicted 13.1% of the variance in job performance at step 1 (*F*_(2, 35)_ = 2.64, *p* = 0.09), MSQ was found to be a significant positive predictor of job performance (β_MSQ_ = 0.35, *t* = 2.13, *p* < 0.05). At step 2, trait EI was found to be a significant positive predictor of job performance, over and above job attitude variables (β_trait EI_ = 0.39, *t* = 2.56, *p* < 0.05). Trait EI predicted a significant 14.0% of unique variance in job performance after controlling for job attitude variables (*F*_Change (1, 34)_ = 6.55, *p* < 0.05).

For Teachers, the model predicted 27.5% of the variance in job performance at step 1 (*F*_(2, 63)_ = 11.92, *p* < 0.05), OCQ was found to be a significant positive predictor of job performance (β_OCQ_ = 0.38, *t* = 3.10, *p* < 0.05). At step 2, trait EI was not a significant predictor of job performance, and OCQ remain a significant predictor of job performance (β_OCQ_ = 0.38, *t* = 3.09, *p* < 0.05). Trait EI predicted 2.8% of unique variance in job performance after controlling for job attitude variables (*F*_Change (1, 62)_ = 2.45, *p* = 0.12).

### Trait emotional intelligence profiles across professions in Kuwait

ANOVA and MANOVA are robust and the sample size is sufficiently large. The analyses were carried out with the seven professions as levels of the independent variable and global and four trait EI factors scores as the dependent variable for ANOVA and MANOVA, respectively.

Levene’s tests of equality of variances for the global trait EI score and the four trait EI factor were nonsignificant, suggesting that the homogeneity of variance assumption was met in all cases.

The main effect on global trait EI was statistically significant (*F*(6, 307) = 4.47, *p* < 0.001, *η*^2^ = 0.080). Tukey HSD *post-hoc* analysis revealed that Military scored significantly lower than all other professions.

There was a significant multivariate main effect of professions on the four trait EI factors (namely, Wellbeing, Self-control, Sociability, and Emotionality), *F*_(24_, _1,228)_ = 2.25, *p* < 0.05, *η_p_*^2^ = 0.04. Follow-up ANOVAs revealed significant differences on three of the four trait EI factors: Wellbeing, *F*_(6,307)_ = 3.95, *p* < 0.001, *η_p_*^2^ = 0.07; Sociability, *F*_(6,307)_ = 4.01, *p* < 0.001, *η_p_*^2^ = 0.07; and Emotionality, *F*_(6,307)_ = 4.97, *p* < 0.001, *η_p_*^2^ = 0.09. While Self-control scores were not statistically different across professions, *F*(6, 307) = 1.91, *p* = 0.08.

Tukey’s *post-hoc* tests indicated that Military, overall, scored significantly lower than all other professions on three trait EI factors (*viz.*, Wellbeing, Sociability, and Emotionality). Notably, Military scored lower than other professions on the fourth trait EI factor (i.e., Self-control), although there was no main effect of professions on Self-control. Furthermore, Lawyers scored significantly higher Sociability scores than Engineers. All other differences were nonsignificant between professions.

## Discussion

The present study set out to investigate trait EI profile differences among professionals in Kuwait. It also assessed the construct’s incremental validity in predicting job performance over and above job attitude variables (i.e., job satisfaction and organizational commitment). Last, it endeavored to parse the relationship between trait EI and performance *via* a mediational pathway through job attitudes.

### The relationship between trait emotional intelligence and job-related variables

We presented the zero-order correlations between the TEIQue-SF variables and the three job-related variables: job performance, job satisfaction, and organizational commitment. As expected from earlier meta-analytic studies (e.g., O'Boyle et al., 2011; Miao et al., 2017), trait EI was significantly correlated with all job-related variables included in our study. Thus, providing another support for the criterion validity of the scores obtained from our Kuwaiti adapted TEIQue-SF (Hasan et al., 2023).

The causal relationships between the key variables in our study were presented earlier in the introduction. Based on meta-analytic studies, the literature showed that trait EI was a significant predictor of job performance (O'Boyle et al., 2011) and job attitudes including job satisfaction and organizational commitment (Miao et al., 2017). Furthermore, Riketta (2008) concluded that job attitudes affected job performance. Therefore, we build our mediation model based on the previous findings, in which we wanted to examine whether the trait EI effect on job performance will be mediated by job attitudes. We did not find any supporting literature for the fact that job attitudes affect trait EI, and therefore, we did not test an alternative model that trait EI will mediate the well-established relationship between job attitudes and job performance.

Our SEM results showed that job attitudes defined by the two indicators of job satisfaction and organizational commitment, partially mediated the relationship between trait EI and job performance. This finding accords with the earlier investigation by Li et al. (2018), who reported that job satisfaction partially mediated the relationship between trait EI and job performance on a large sample of teachers in China. The key difference between our study and their study is the role of job satisfaction variable in the mediation model. In their study, they treated job satisfaction as a key variable in the model (i.e., a mediator variable). While in our study, job satisfaction was modeled together with organizational commitment as an indicator of a general factor of job attitudes. Even though the two studies reached the same conclusions, we believe that further confirmations in longitudinal designs are desirable.

### Incremental validity of trait emotional intelligence

Another aim of this study was to assess the incremental validity of trait EI (measured by the Kuwaiti-Arabic TEIQue-SF) in predicting job performance. This is one of a small number of studies to assess the incremental validity of TEIQue in predicting job performance across different professions (e.g., Petrides et al., 2022).

Our results showed that global trait EI incrementally predicted job performance over job attitudes in Policemen and Engineers. In contrast, no such effects were observed in other professions (e.g., Bankers, Healthcare providers, Lawyers, Military, and Teachers). However, this result should not be taken as conclusive owing to the relatively small sample sizes for this kind of analysis.

A limitation in our research design is using a single item to measure job performance as a criterion variable. On the one hand, Fuchs and Diamantopoulos (2009) suggested using a single-item scale if the construct is referring to a concept that received a global consensus on what does it mean (i.e., concrete construct), if the population is diverse, and if the sample size is relatively small. On the other hand, Credé et al. (2012) argued that using abbreviated measures (e.g., 1-item) will affect our estimation of the relationship between personality traits with other behavioral constructs. Accordingly, the relationship between trait EI and job performance may be greater than suggested in our study. Taking into account both views, we used a single-item scale for job performance because we believe that: (1) the concept is concrete; (2) our sampling population is highly diverse (e.g., several professions); and (3) the sample size is small for such design.

Another limitation of our study is the use of self-reported measures. One problem with this type of measure is related to the participant’s responses in which they respond in a socially acceptable way (i.e., social desirability bias) or in a certain way regardless of the question (i.e., response bias). Another problem is related to the clarity of the items to the participants, which can lead to different interpretations of the questions. However, we tried to minimize these biases by including well-established measures in Kuwait.

At any rate, future research could rectify these limitations through the use of longer measures or objective job performance criteria with larger samples.

### Trait emotional intelligence profiles across professions in Kuwait

We also aimed to compare the trait EI profiles (global and four-factor scores) across different professions in Kuwait. Our analysis suggested that the Military had lower scores on global trait EI and on three of four factors (Self-control being the exception where scores were numerically but not statistically lower). Although the differences were insignificant on the Self-control factor, they still scored numerically lower than other professions. Due to the serious consequences of potentially emotionally driven decision-making within the military sector, it is concerning that members of the military profession in our study had lower trait EI scores. Unfortunately, several reports from Kuwait reported a crime that involves a person working in the Military sector (e.g., an army person shot and killed his colleague in an army camp, Ibrahim, 2022).

In fact, the Kuwaiti Military participants in our study scored lower than their counterparts in the United States (Bond, 2016; Walters, 2018; Placek et al., 2019), the United Kingdom (MacEwan and Gibson, 2022; Petrides et al., 2022), and France (Bourgeon et al., 2015). This result was not surprising for us because psychological and personality-related tests are considered in either selection processes or after their employment in the United States (e.g., Global Assessment Tool), in the United Kingdom (e.g., Threshold Assessment Grid), and in France (e.g., Psychotechnical tests).

These findings are important for the Military sector in Kuwait as their employees may benefit from trait EI training programs. Because trait EI can be changed through training, as previous research has shown (for details, Nelis et al., 2009; Petrides et al., 2016). In fact, it is helpful to optimize trait EI in all employees, as it affects their general wellbeing, health, social relationships, and work performance (Mavroveli et al., 2007; Mikolajczak et al., 2009; Li et al., 2018; Sarrionandia and Mikolajczak, 2020).

Lawyers scored significantly higher than Engineers on the Sociability factor and numerically higher than all other professions. However, we believe that the result of our study is not surprising. This is because the Sociability factor emphasizes social relationships and social influence in the workplace in this case. Petrides (2009) indicated that higher scores on the Sociability factor mean that the individual has good listening skills and can communicate with others. We believe that these two characteristics are important for Lawyers as their work is based on interactions with others (Gerdy, 2013). While for Engineers, the nature of their profession may prevent them from these interactions as they mostly deal with numbers and machines.

We believe that most professions could benefit from trait EI scores as it affects other job-related variables, as shown in the current study and the wider literature. For example, Military should have higher Self-control scores as it is important in this line of work to able to regulate external pressures and stress. Actually, in a similar line of work, Dugger et al. (2022) found that Pilots did not score lower than the US general population on the Self-control factor of trait EI. What is more, the trait EI profiles in certain professions may indirectly affect other individuals. For instance, the teacher’s trait EI affects students’ academic performance (Chamizo-Nieto et al., 2021; Perez Diaz, 2021). Consequently, we call on employers to pay more attention to their employees’ trait EI and to offer the appropriate training for the best outcomes.

To our knowledge, psychological or personality testing is not considered for prospective soldiers by the Kuwaiti Military sector (or any other governmental sector). Even more, their admission requirements focus on physical fitness and health (generally, diploma, and qualifications in other sectors), but not on their mental health using different psychological and personality assessments. Even after graduating from the Military Academy in Kuwait, Military leaders are not considering any psychological or emotional training for their soldiers. As reported by the Kuwait News Agency (Aldeqbasi, 2019), the focus is on physical and military-related training such as Special Weapons and Tactics (SWAT) training. Unfortunately, this is true in other sectors, as well.

Furthermore, this study explored the trait EI profiles of several professions such as Policemen, Military, Teachers, Healthcare providers, Bankers, Engineers, and Lawyers in Kuwait. Trait EI scores in certain professions (e.g., Military) were notably lower than others indicating a need for employee trait EI training because higher trait EI scores affect several important job-related variables such as job performance and job attitudes. One potential reason for the low trait EI scores in certain professions is that their emotions are not acknowledged and treated with respect.

Smollan and Sayers (2009) argued that respecting employee’s emotions by the organization will positively influence the employee’s organizational commitment and their reactions toward everyday’s job events. Consequently, we encourage employers not only to focus on the job-related qualifications and degrees of their prospective employees but also on their personality traits and, more importantly, on their trait EI. This is because Sackett et al. (2021) showed that trait EI was the first non-job-related variable (i.e., personality-related) predictor for job performance.

## Conclusion

Notwithstanding the aforementioned limitations of this study, we believe that it is crucial as it is one of the first attempts to study trait EI across different professions in a country that has not been well-represented in the global trait EI literature. This will not only encourage Kuwaiti researchers to explore these trait EI profiles of professionals in Kuwait but also allow for future cross-cultural studies. The present study is also the only empirical investigation into the mediating role of job attitudes in the relationship between trait EI and job performance, in which we showed that trait EI still has a substantial effect on job performance even after presenting another well-established job-related variable into the path model. Although the results are not definitive due to the small sample size, we investigated the incremental validity of trait EI in predicting job performance in several jobs. To our knowledge, this is the first study including this number of different professions.

Lastly, this study throws up many questions that need further investigation. First, researchers should consider a longitudinal design to test our proposed mediation model. Second, the incremental validity of TEIQue-SF should be assessed using a relatively more comprehensive validated job-related measure. Third, future researchers may consider the effects of trait EI training on both trait EI profiles and job performance. And more importantly, researchers should consider non-self-report measures as criteria to assess the relationships presented in our study.

## Data availability statement

The raw data supporting the conclusions of this article will be made available by the authors, without undue reservation.

## Ethics statement

The studies involving human participants were reviewed and approved by the University College London-Departmental Ethics Committee (CEHP/2021/586). The patients/participants provided their written informed consent to participate in this study.

## Author contributions

NH and KP conceived the study and conducted the statistical analysis. NH collected the data and wrote the manuscript with revisions and contributions from KP, LH, and FH. NH, KP, LH, and FH designed the study. All authors contributed to the article and approved the submitted version.

## Conflict of interest

The authors declare that the research was conducted in the absence of any commercial or financial relationships that could be construed as a potential conflict of interest.

## Publisher’s note

All claims expressed in this article are solely those of the authors and do not necessarily represent those of their affiliated organizations, or those of the publisher, the editors and the reviewers. Any product that may be evaluated in this article, or claim that may be made by its manufacturer, is not guaranteed or endorsed by the publisher.
